# The prognosis of lipid reprogramming with the HMG-CoA reductase inhibitor, rosuvastatin, in castrated Egyptian prostate cancer patients: Randomized trial

**DOI:** 10.1371/journal.pone.0278282

**Published:** 2022-12-08

**Authors:** Riham M. Karkeet, Abdelrahman N. Zekri, Mohamed M. Sayed-Ahmed, Ghada M. Sherif, Salem E. Salem, Ahmed Abdelbary, Mariam A. Fouad, Sherif Y. Saad

**Affiliations:** 1 Pharmacology and Experimental Oncology Unit, Cancer Biology Department, National Cancer Institute, Cairo University, Cairo, Egypt; 2 Virology and Immunology Unit, Cancer Biology Department, National Cancer Institute, Cairo University, Cairo, Egypt; 3 Biostatistics and Cancer Epidemiology Department, National Cancer Institute, Cairo University, Cairo, Egypt; 4 Medical Oncology Department, National Cancer Institute, Cairo University, Cairo, Egypt; 5 Department of Surgical Oncology, National Cancer Institute, Cairo University, Cairo, Egypt; 6 Moleculat Therapeutics Program, Fox Chase Cancer Center, Philadelphia, PA, United States of America; PhD, PLOS, UNITED KINGDOM

## Abstract

**Aim:**

The role of surgical castration and rosuvastatin treatment on lipid profile and lipid metabolism related markers was evaluated for their prognostic significance in metastatic prostate cancer (mPC) patients.

**Methods:**

A total of 84 newly diagnosed castrated mPC patients treated with castration were recruited and divided into two groups: Group I served as control (statin non-users) while group II treated with Rosuvastatin (20 mg/day) for 6 months and served as statin users. Prostate specific antigen (PSA), epidermal growth factor receptor (EGFR), Caveolin-1 (CAV1), lipid profile (LDL, HDL, triglycerides (TG) and total cholesterol (TC)) and lipid metabolism related markers (aldoketoreductase (AKR1C4), HMG-CoA reductase (HMGCR), ATP-binding cassette transporter A1 (ABCA1), and soluble low density lipoprotein receptor related protein 1 (SLDLRP1)) were measured at baseline, after 3 and 6 months. Overall survival (OS) was analyzed by Kaplan-Meier and COX regression for prognostic significance.

**Results:**

Before castration, HMG-CoA reductase was elevated in patients <65 years (P = 0.009). Bone metastasis was associated with high PSA level (P = 0.013), but low HMGCR (P = 0.004). Patients with positive family history for prostate cancer showed high levels of EGFR, TG, TC, LDL, alkaline phosphatase (ALP), but low AKR1C4, SLDLRP1, CAV1 and ABCA-1 levels. Smokers had high CAV1 level (P = 0.017). After 6 months of castration and rosuvastatin administration, PSA, TG, LDL and TC were significantly reduced, while AKR1C4, HMGCR, SLDLRP1, CAV1 and ABCA-1 were significantly increased. Overall survival was reduced in patients with high baseline of SLDLRP1 (>3385 pg/ml, P = 0.001), PSA (>40 ng/ml, P = 0.003) and CAV1 (>4955 pg/ml, P = 0.021).

**Conclusion:**

Results of the current study suggest that the peripheral lipidogenic effects of rosuvastatin may have an impact on the treatment outcome and survival of castrated mPC patients.

**Trail registration:**

This trial was registered at the Pan African Clinical Trial Registry with identification number PACTR202102664354163 and at ClinicalTrials.gov with identification number NCT04776889.

## Introduction

Globally, prostate cancer (PC) represents the second most frequently diagnosed malignant disease and the sixth leading cause of cancer related death among men and its burden is almost expected to be doubled by 2040 due to population growth and aging [1]. In Egypt, PC represents 4.5% of male malignancies, but with apparently low incidence and high mortality due to higher prevalence of advanced and metastatic disease [2, 3]. The treatment of advanced PC is basically dependent on the initial effective responses with androgen deprivation therapy (ADT), through which patients’ progress over time to metastatic castration-resistant prostate cancer (mCRPC). Strategies developed to counteract ADT resistance have had only modest clinical benefit [4, 5]. Nevertheless, relapsed or metastatic disease following castration has a poor prognosis with most patients dying within two years [6–10].

The metabolic effects of ADT, including disturbance of lipid profile leading to increased risk for diabetes, metabolic syndrome and cardiovascular morbidity/mortality may have a role in treatment related morbidity in these patients [11]. Lipid metabolism is strikingly affected by androgens and dysregulation of lipid metabolism is a key feature of PC development. Androgens may stimulate *de novo* lipogenesis and lipid uptake. HMG-CoA reductase inhibitors (Statins) are administered widely worldwide for their clinical expressive lipid-lowering effect. Statins not only prevent the production of cholesterol, but also block the formation of many intermediary lipid molecules, which might function as cellular signaling pathways stimulant, provoking the major function of cholesterol synthesis in accelerating progression to CRPC.

Several epidemiological reports have shown significant associations between statin use and decreased incidence of advanced PC, risk of recurrence after local treatment, mortality, and PSA levels relative to Statin nonusers [12, 13]. Statins decrease advanced stage prostate cancer risk raising a possible correlation between cholesterol and prostate cancer risk [14]. Moreover, high cholesterol levels have proved to increase the risk of aggressive PC, and according to Jiang and colleagues, statin users after prostatectomy had a less aggressive disease [15]. Rosuvastatin is the most powerful widely‐prescribed statin worldwide; due to its rapid absorption, achieving peak plasma concentration within three hours. Moreover, its lipid‐lowering effect is not influenced by the time‐of‐day by which it is administered; due to the comparatively long half‐life of 20 hours [16, 17]. Accordingly, the current study has been initiated to investigate, on mechanism-based, the possible therapeutic effects of rosuvastatin when combined with androgen deprivation in metastatic prostate cancer patients.

## Patients, materials and methods

### I. Study design

This is a prospective un-blinded randomized controlled study, including a control group (Group I) and an intervention group (Group II). Parallel design was used with 1:1 allocation. The study ID BB1901-30303 was approved by the Institutional Human Research Ethics Committee of NCI, Egypt, Number 00004025, with IRB review Number 201819019.3 and conducted in accordance with the Declaration of Helsinki with written informed consent taken from all participants. This trial was registered at the Pan African Clinical Trial Registry with identification number PACTR202102664354163 and at ClinicalTrials.gov with identification number NCT04776889; after starting the enrolment of participants as the Institute mandates only IRB approval. The authors confirm that all ongoing and related trials for this intervention are registered.

### II. Participants

**Fig 1:**
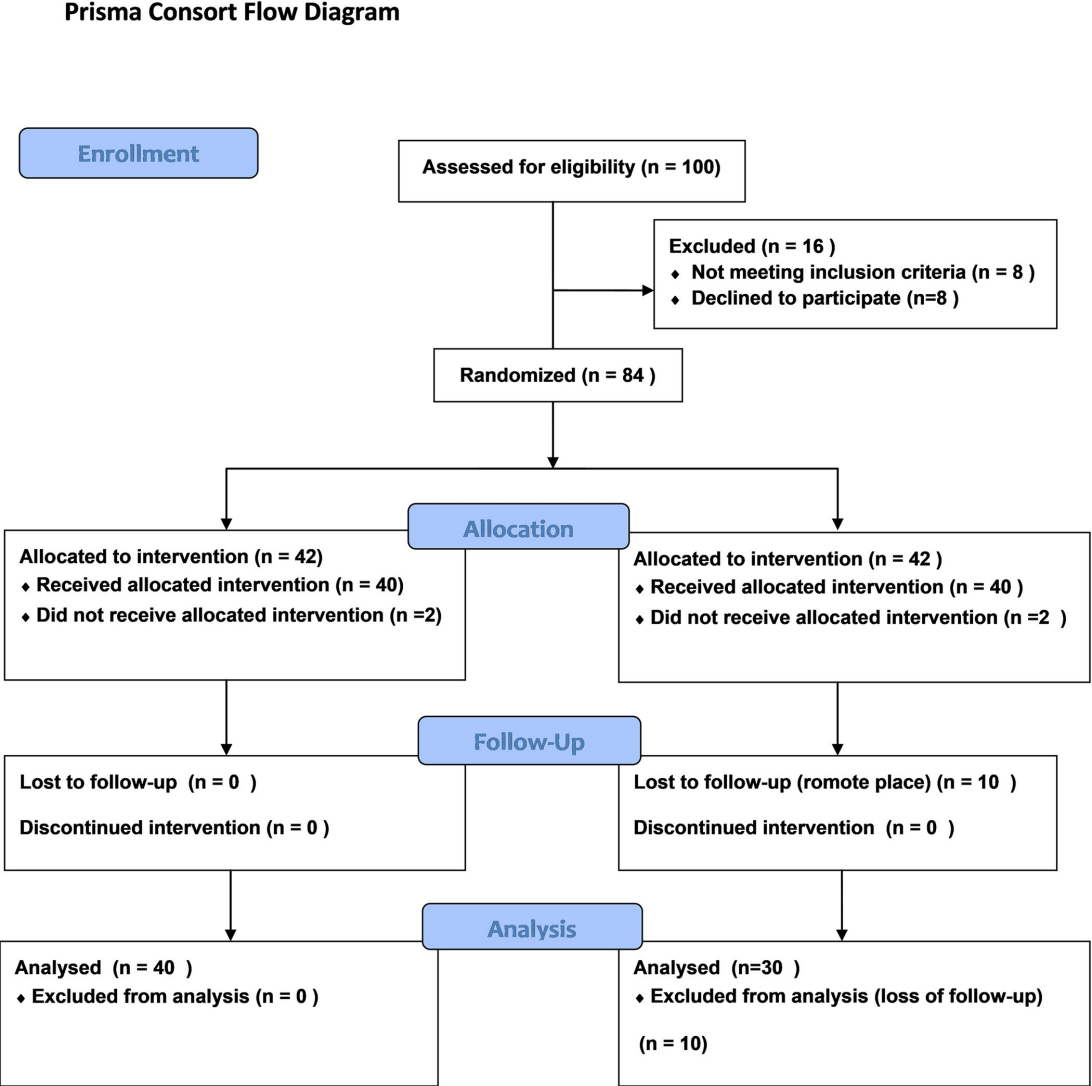
Prisma consort flow diagram.

A cohort of 84 newly diagnosed metastatic prostate cancer patients were recruited at the National Cancer Institute (NCI), Cairo University according to the eligibility criteria of being naïve newly diagnosed with metastatic prostate cancer, aged ≥ 50 years and with no psychological or geographical barriers for regular follow up. Patients were recruited from 15^th^ January to 30^th^ June 2019, then followed-up to 30^th^ December 2020. Patients were randomized into 2 equal groups (Fig 1).

### III. Interventions

The treatment modality and decision making were according to NCI guidelines. Surgical castration in the form of bilateral subcapsular orchiectomy was deemed clinically necessary for all trial participants as decided by the treating team. Group I served as control (statin non-users). Group II treated with Rosuvastatin (20 mg/day) for 6 months and served as statin users.

### IV. Outcomes

Primary outcome was assessing the change in molecular parameters at 3 and 6 months compared to baseline levels; while secondary outcome was assessing the short term clinical and biochemical response in terms of disease progression/regression and overall survival.

Blood samples were withdrawn from all patients at baseline to monitor parameters under investigation. Blood samples were then withdrawn from the two groups 3 and 6 months after castration. Full demographic information and clinicopatholologic characteristics were obtained for each patient including; age, comorbid diseases, initial complain symptoms, family history of malignancy, smoking status and performance status and were summarized in **Table 1**. Also, serum level of prostate specific antigen (PSA) and alkaline phosphatase (ALP), Gleason score and metastatic sites were recorded. Ten patients from the control group lost follow-up and were excluded from all tests.

**Table 1:** Demographic and clinicopathological characteristics of metastatic prostate cancer patients.

		Count	%	Control	Treated
Total		70	100%	30	40
**Age groups**	<65 years	22	31.4%	8	14
	65–75 years	42	60.0%	18	24
	>75 years	6	8.6%	4	2
**Baseline PSA**	<10	2	2.9%	2	0
	= 10.1–20	4	5.7%	0	4
	= 20.1–40	10	14.3%	2	8
	= 40.1–99	8	11.4%	6	2
	>100	46	65.7%	20	26
**Bone metastasis**	Negative	6	8.6%	2	4
	Positive	64	91.4%	28	36
**Lung metastasis**	Negative	50	71.4%	34	26
	Positive	20	28.6%	6	14
**Palliative radiotherapy**	Negative	16	22.9%	6	10
	Positive	54	77.1%	24	30
**Family history**	Negative	60	85.7%	24	36
	Positive	10	14.3%	6	4
**Comorbidities**	Negative	38	54.3%	20	18
	Positive	32	45.7%	10	22
**Hypertension**	Negative	48	68.6%	20	28
	Positive	22	31.4%	10	12
**Diabetes Mellitus**	Negative	52	74.3%	24	28
	Positive	18	25.7%	6	12
**Cardiac**	Negative	60	85.7%	28	32
	Positive	10	14.3%	2	8
**Smoking**	Negative	12	17.1%	4	8
	Positive	58	82.9%	26	32
**Gleason scoring**	≤7	28	40%	12	16
	= 8–10	42	60%	18	24
**Bone pain grading**	Mild to moderate	36	51.4%	14	22
	Severe	34	48.6%	16	18
**Baseline ALP**	44–147 IU/L (normal)	22	31.4%	8	14
	> 147 (high)	48	68.6%	22	26
**Performance status**	1	30	42.80%	13	17
	2	20	28.57%	9	11
	3	14	20.00%	6	8
	4	6	8.57%	2	4
**Baseline LDL**	<100 mg/dl (normal)	16	22.86%	8	8
	>100 mg/dl (high)	54	77.14%	22	32
**Baseline TG**	<150 mg/dl (normal)	28	40.0%	12	16
	> 150 (high)	42	60.0%	18	24
**Baseline cholesterol**	<170 mg/dl (normal)	24	34.3%	10	14
	> 170 (high)	46	65.7%	20	26
**Baseline HDL**	>45 mg/dl (normal)	40	57.1%	20	24
	< 45 (low)	30	42.8%	10	16
**Status**	Alive	52	74.3%	19 (63.4%)	33 (82.5%)
	Dead	18	25.7%	11(36.6%)	7 (17.5%)
**Response**	Regression	48	68.6%	20 (66%)	28(70%)
	Progression	22	31.4%	10 (33%)	12 (30%)

### V. Sample size determination

Sample size was calculated based on the previous paper by Gann and colleagues, 2001 [18], the expected difference between the 2 groups in EGFR level change from baseline to 6 months was about 35%. Using the Kelsey method with (power 85% and 5% significance level), 35 patients were required in each group. This number was increased to 42 in each group to overcome a potential drop out of 15%. Sample size calculation was achieved using PS: Power and Sample Size Calculation software Version 3.1.2 (Vanderbilt University, Nashville, Tennessee, USA).

### VI. Randomization, allocation and masking

Patients were randomly allocated using closed envelop method into one of the two study groups. For allocation of participants, a computer-generated list of random numbers was used following block randomization procedure with block size of 4. Recruitment was performed by investigators A.A. and S.E.S. Randomization was prepared by the trial statistician. Rosuvastatin was dispensed to the patients from the outpatient pharmacy after prescription by the trial oncologist. The study was unblinded as both the patients and the treating physician knew the allocated treatment. However, the study statistician performing the final analysis was masked to interventions.

### VII. Statistical methods

Statistical analyses were performed using The Statistical Package for Social Sciences (SPSS) version 24. Normal distribution and variance homogeneity of data were assessed using the Kolmogorov–Smirnov and Levene’s tests, respectively. Numerical data were summarized using median and interquartile range (IQR). Categorical data were summarized as count and percentage. Patients were stratified according to their clinic-pathological factors and for more than two subgroups of patients, the change in measured parameters were tested for significance using Kruskal–Wallis test and the pairwise comparison were done using Mann–Whitney. The change in proteins concentration over time was tested using Friedman test of significance. Spearman correlation analysis was used to test all possible correlations. Kaplan- Meier survival analysis was used to calculate the cumulative survival rate as well as median levels of OS after two years of follow up. OS was calculated from date of diagnosis to date of death by any cause. Living patients or patients lost to follow-up were censored on the last known alive date. The hazardous effect of death or progression Cox proportion hazard Model was used to evaluate the hazardous effect of different clinicopathological and proteins levels on death and progression. All P-values are two-sided. P-values < 0.05 were considered significant.

### VIII. Chemicals

ELISA kits for human epidermal growth factor receptor (EGFR), Caveolin-1 (CAV1), lipid profile (LDL, HDL, TG and TC) and lipid metabolism related markers (AKR1C4, HMGCR, ABCA1, and SLDLRP1) were supplied from SUNLONG BIOTECH CO., LTD, Hangzhou, Zhejiang, China.

### IX. Laboratory methods

Collected whole blood samples into k.EDTA tubes were incubated at room temperature for 15 minutes and then centrifuged for 20 minutes at 1500 rpm. The plasma supernatant was carefully collected and freezed until analysis. Plasma protein concentrations of some lipid reprogramming and prostate cancer aggressiveness markers were measured. Kits were probed against human soluble low density lipoprotein receptor related protein-1 (SLDLRP1; catalogue number: SL2705Hu), human ATP binding cassette transporter A-1 (ABCA-1; catalogue number: SL0314Hu), Human Aldoketo-reductase family 1 member C4 (AKR1C4; catalogue number: SL3032Hu), human 3-hydroxy-3-methylglutaryl Co-enzyme A reductase (HMGCR; catalogue number: SL3030Hu), human epidermal growth factor receptor (EGFR; catalogue number: SL0665Hu) and human Caveolin-1 (CAV1; catalogue number: SL0427Hu). Procedures were carried out in accordance with the manufacturer’s instructions. The concentration of the markers in plasma samples was calculated by comparing the optical density (OD) of the samples to the corresponding plotted standard curves.

## Results

### The demographic and clinicopathological characteristics

Urine retention and bone aches were the most frequent presenting complaints. Most of cases (42.8%) had good performance status (ECOG I), although Gleason scoring was 8–10 in (61.8%) and PSA > 100 in (65.7%).

### Rosuvastatin suppresses lipid profile in mPC patients (Fig 2)

**Fig 2:**
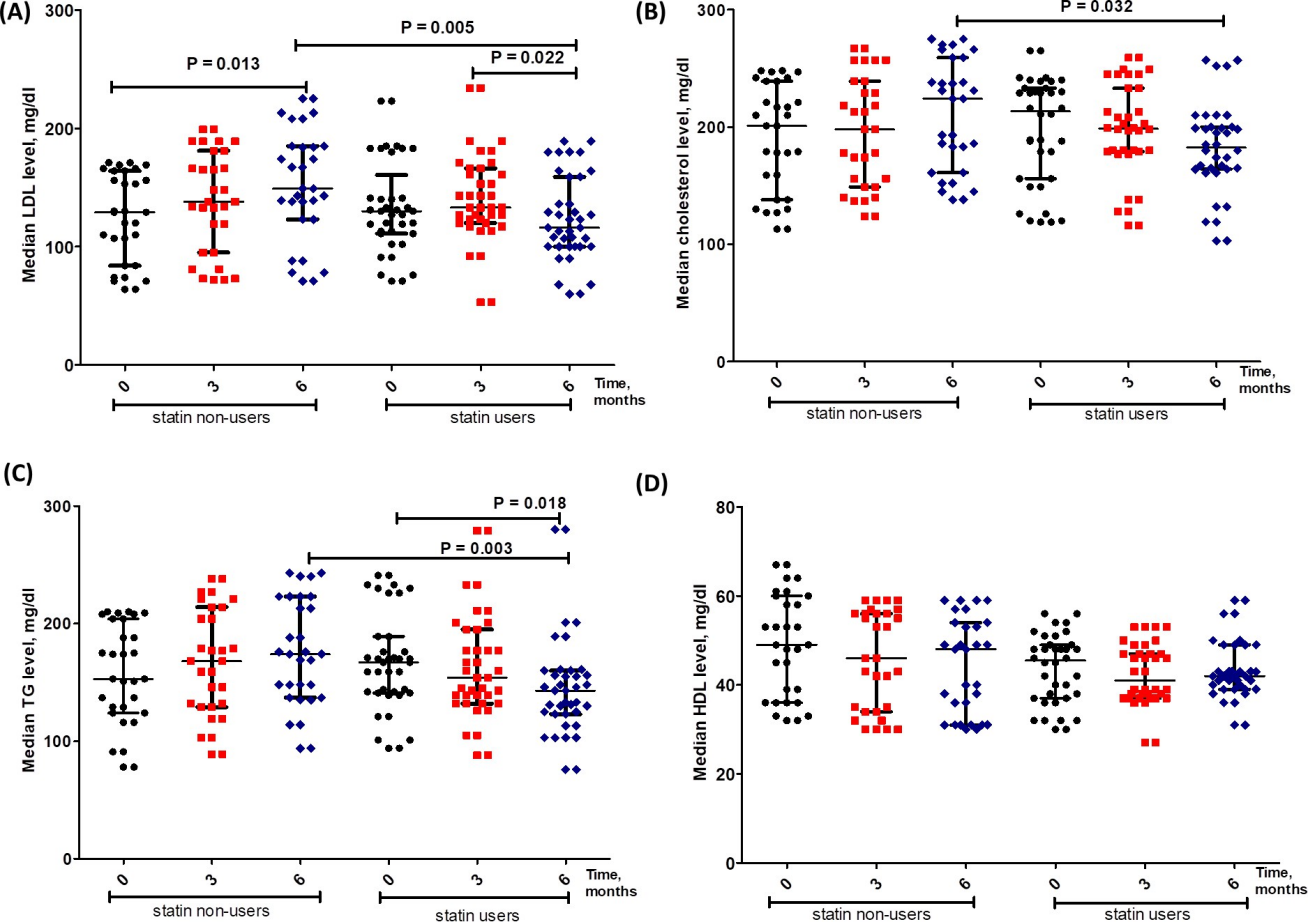
Levels of LDL (A), TC (B), TG (C) and HDL (D) at baseline, after 3 and 6 months of castration in statin and non-statin users PC patients. Data are presented as median levels of the tested markers in control and treated PC patients.

Fig 2 shows the changes in the levels of lipid profile including LDL (A), TC (B), TG (C) and HDL (D) at baseline, after 3 and 6 months of castration in statin and non-statin users PC patients. Six months after castration and Rosuvastatin treatment, the levels of LDL, cholesterol and TG were significantly decreased in statin-treated group as compared to non-statin users (p = 0.005, 0.032 and 0.003, respectively). In the same context, the statin users group recorded around 20% lower median levels of lipid profile parameters as compared to non-statin users group. Also, statin non-users group showed a significant increase in LDL level after 6 months of castration as compared to the base line (p = 0.013). However, non- significant changes were detected in HDL levels either within or between statin and non-statin users patients.

### Rosuvastatin increases the levels of lipid metabolism-related proteins (Fig 3)

**Fig 3:**
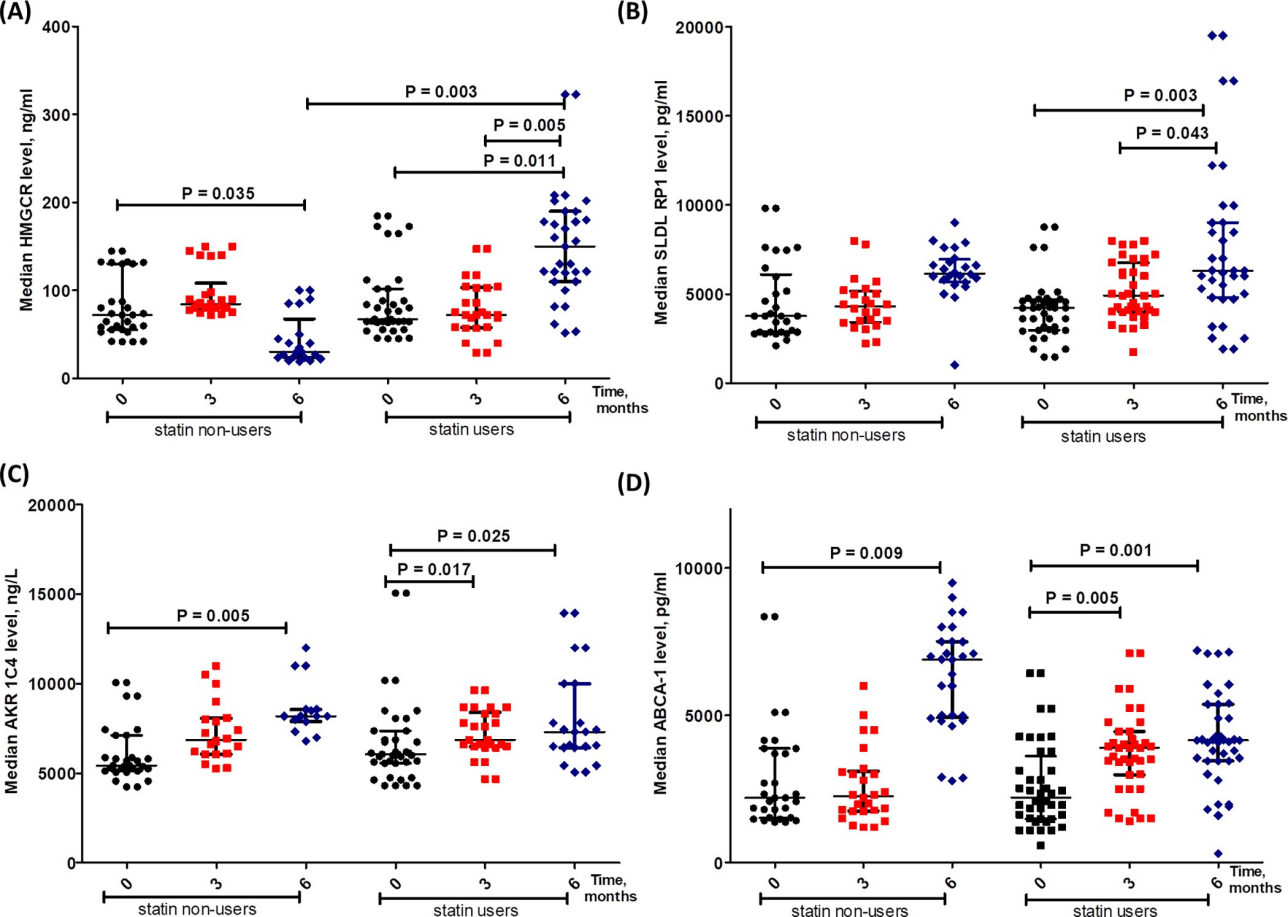
Levels of HMGCR (A), SLDLRP1 (B), AKR1C4 (C) and ABCA-1 (D) at baseline, after 3 and 6 months of castration in statin and non-statin users PC patients. Data are presented as median levels of the tested markers in control and treated PC patients.

To investigate the potential prognostic significance of lipid metabolism-related proteins following surgical castration and Rosuvastatin treatment, we measured the levels of HMGCR, SLDLRP1, AKR1C4 and ABCA-1 at baseline, after 3 and 6 months of castration in statin and non-statin users PC patients. A significant difference was observed in HMGCR levels between the 2 groups after 6 months of castration with 78% higher median level in statin users at p = 0.003 (Fig 3A). In statin users group, the level of SLDLRP1 (Fig 3B) after 6 months was significantly higher when compared to their base-line and 3 months levels (p = 0.003 and 0.043). In both statin and non-statin users groups, AKR1C4 levels were significantly elevated at 6 months when compared to their baseline values at p = 0.025 and 0.005 (Fig 3C) and non-significant changes were observed between the two groups. Similarly, in both statin and non-statin users, the levels of ABCA-1 showed a significant increase after 3 and 6 months of castration as compared to their baseline values at p = 0.001 and 0.009, respectively (Fig 3D).

### Differential changes in the levels of PSA, CAV1 and EGFR between statin users and non-users mPC patients (Fig 4)

**Fig 4:**
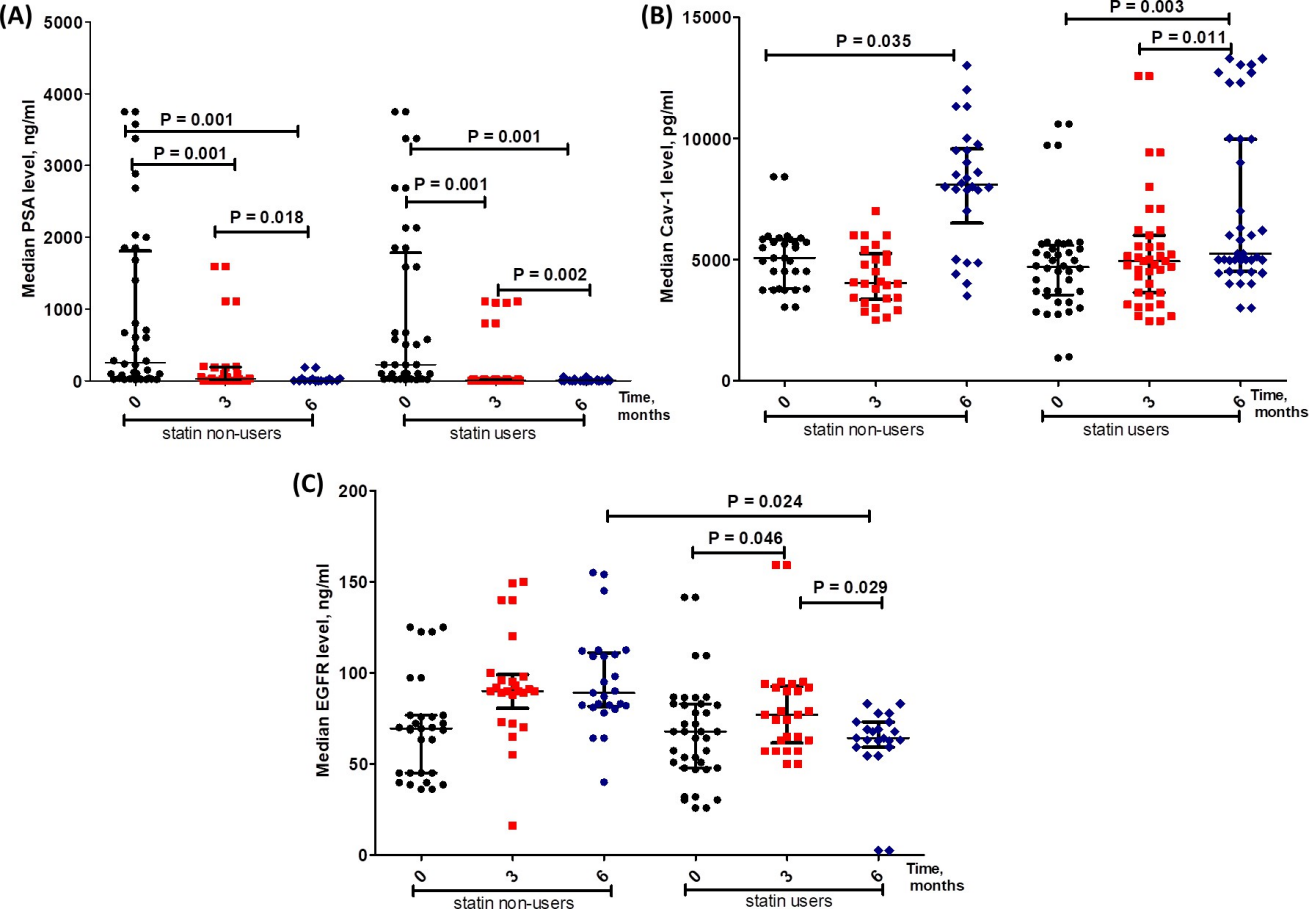
Levels of PSA (A), Caveolin-1 (B) and EGFR (C) at baseline, after 3 and 6 months of castration in statin and non-statin users PC patients. Data are presented as median levels of the tested markers in control and treated PC patients.

To test the effects of Rosuvastatin treatment on the outcome of prostate cancer, we monitored the levels of PSA, CAV1 and EGFR at baseline, after 3 and 6 months in statin and non-statin users PC patients. As shown in Fig 4A, the median level of PSA showed marked and significant decrease at 3 and 6 months as compared to the baseline level in both statin and non-statin users groups (p = 0.001) although non-significant changes were observed between the two groups at all-time points. On the other hand, CAV1 level showed a significant increase of 36% (p = 0.035) at 6 months compared to baseline in the non-statin user group compared to a modest 9.5% increase in statin users (p = 0.003) (Fig 4B). Although there is a consistent trend of EGFR upregulation in the control (non-statin user) group, in statin users group, EGFR level was significantly increased at 3 months compared to the base line value (p = 0.046), but significantly decreased by 22% at 6 months (p = 0.024) as compared to non-statin users group (Fig 4C).

### Correlation between baseline levels of lipid profile markers (LDL, Cholesterol, TG and HDL) and mPC patient characteristics (Fig 5)

**Fig 5:**
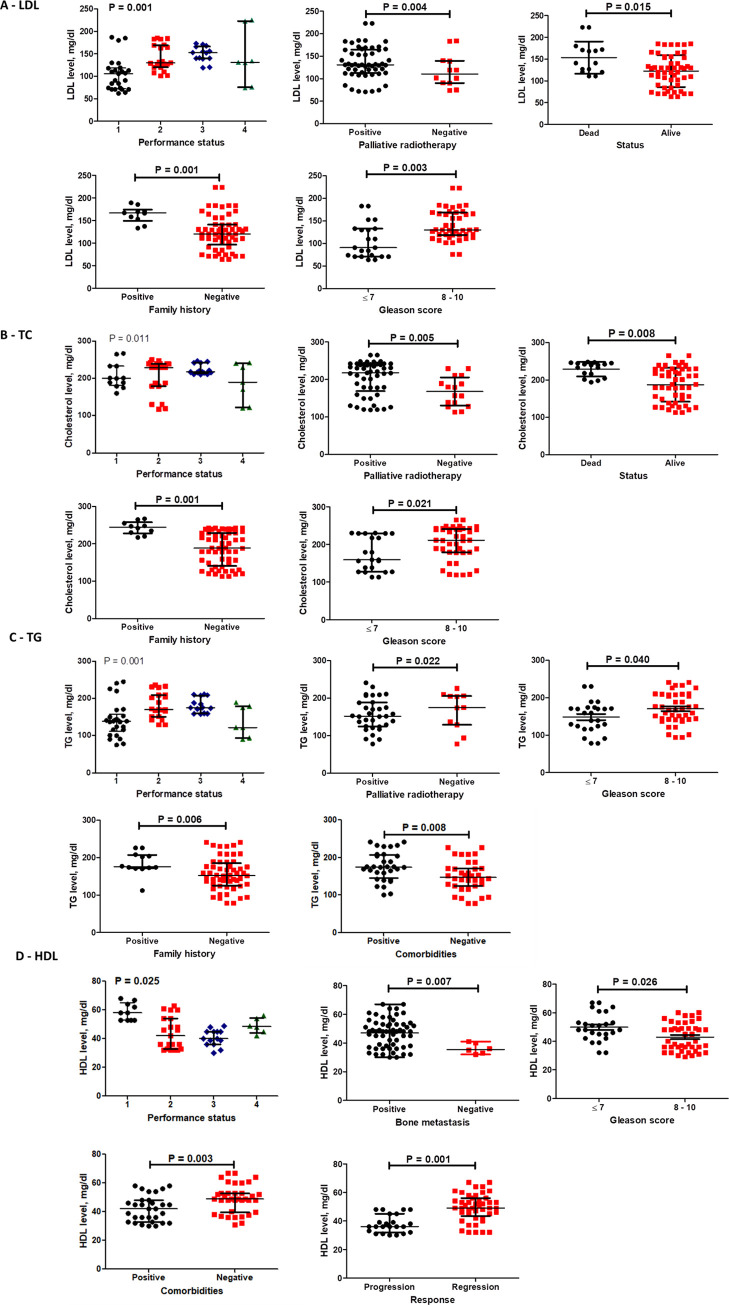
Subgroup analysis for the baseline levels of LDL (A), TC (B), TG (C) and HDL (D). Data are presented as medians and IQR for the baseline levels of listed markers. Significant difference in levels between the subgroups of patients detected using Mann-Whitney for two subgroups and Kruskal Wallis for more than two subgroups.

Higher median LDL level was significantly associated with performance status 3, the need to receive palliative radiotherapy, positive family history, Gleason score >7 and mortality (p = 0.001, 0.004, 0.001, 0.003 and 0.015) (Fig 5A). Similarly, high total cholesterol (TC) median level showed a significant association with palliative radiotherapy, family history, Gleason score >7 and performance status 3 and mortality (p = 0.005, 0.001, 0.021, 0.011 and 0.008) (Fig 5B). Higher median TG level was associated significantly with requiring palliative radiotherapy, positive family history, presence of comorbidities, Gleason score >7 and performance status 4 (p = 0.022, 0.006, 0.008, 0.040 and 0.001) (Fig 5C). In contrast, HDL median level were associated with performance status, Gleason score 7, absence of comorbidities, disease regression and survival (p = 0.025, 0.026, 0.003, 0.001 and 0.016) (Fig 5D). Interestingly, higher levels of HDL were associated with positive bone metastasis (p = 0.007).

### Associations of baseline levels of lipid metabolism related proteins (HMGCR, ABCA-1, AKR1C4 and SLDLRP1) with mPC patient characteristics (Fig 6)

**Fig 6:**
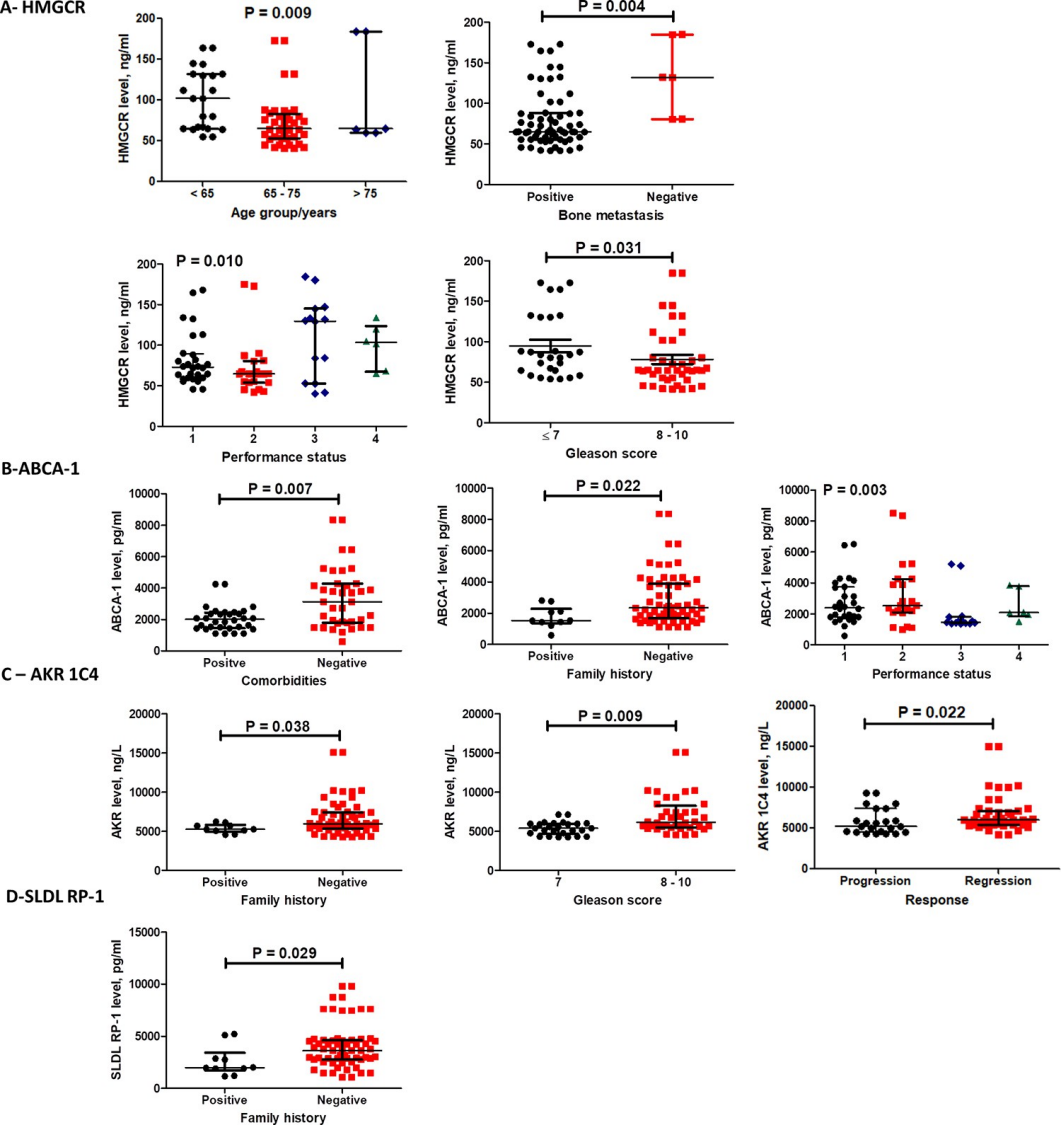
Subgroup analysis of baseline protein levels of HMGCR (A), ABCA-1 (B), AKR1C4 (C) and SLDLRP1 (D). Data are presented as medians and IQR for the baseline levels of listed markers. Significant difference in levels between the subgroups of patients detected using Mann-Whitney for two subgroups and Kruskal Wallis for more than two subgroups.

High median level of HMGCR was significantly associated with the age < 65 years, absence of bone metastasis, Gleason score 7 and performance status 3 (p = 0.009, 0.004, 0.031 and 0.010) (Fig 6A). Furthermore, high median ABCA-1 level was significantly associated with negative family history, absence of comorbidities, performance status 4 and survival (p = 0.022, 0.007, 0.003 and 0.034) (Fig 6B). AKR1C4 higher median level showed a significant association with negative family history, Gleason score > 7 and regressive course of disease (p = 0.038, 0.009 and 0.022) (Fig 6C). Additionally, SLDLRP1 level showed significant association with negative family history (p = 0.029) (Fig 6D).

### Association of baseline protein levels of PSA, ALP, CAV1 and EGFR with mPC patient characteristics (Fig 7)

**Fig 7:**
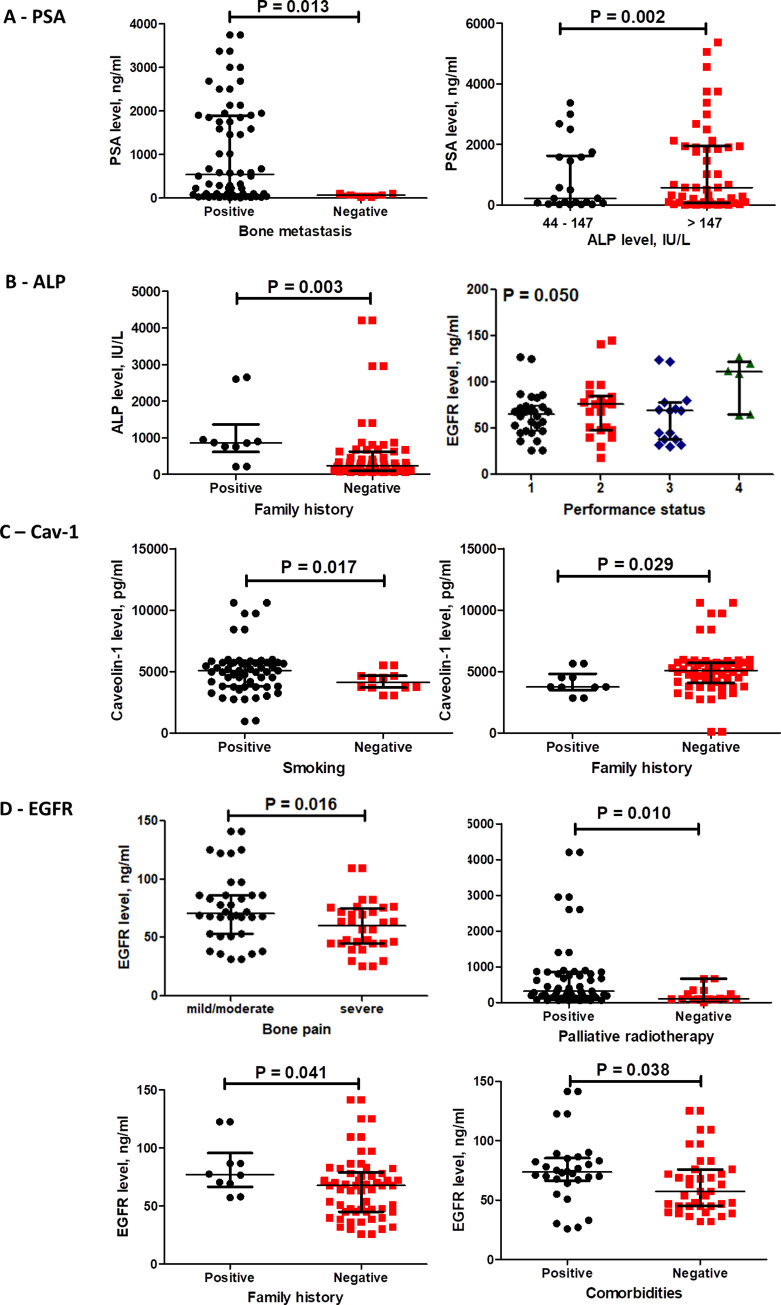
Subgroup analysis of baseline protein levels of PSA (A), ALP (B), Caveolin-1 (C) and EGFR (D). Data are presented as medians and IQR for the baseline levels of listed markers. Significant difference in levels between the subgroups of patients detected using Mann- Whitney for two subgroups and Kruskal Wallis for more than two subgroups.

Fig 7 presents as shown in Fig 7A, the median PSA level was significantly associated with bone metastasis and baseline level of ALP (p = 0.013 and 0.002). Regarding ALP median level it was significantly associated with requirement of palliative radiotherapy, family history and mortality (p = 0.010, 0.003 and 0.029) (Fig 7B). In addition, CAV1 median level was associated significantly with negative family history and smoking (p = 0.029 and 0.017) (Fig 7C). Moreover, EGFR median level was significantly associated with positive family history, comorbidities, and mild to moderate bone pain and performance status 4 (p = 0.041, 0.038, 0.016 and 0.050) (Fig 7D).

### Spearman correlation analysis between LDL with triglycerides and LDL with cholesterol (Fig 8)

**Fig 8:**
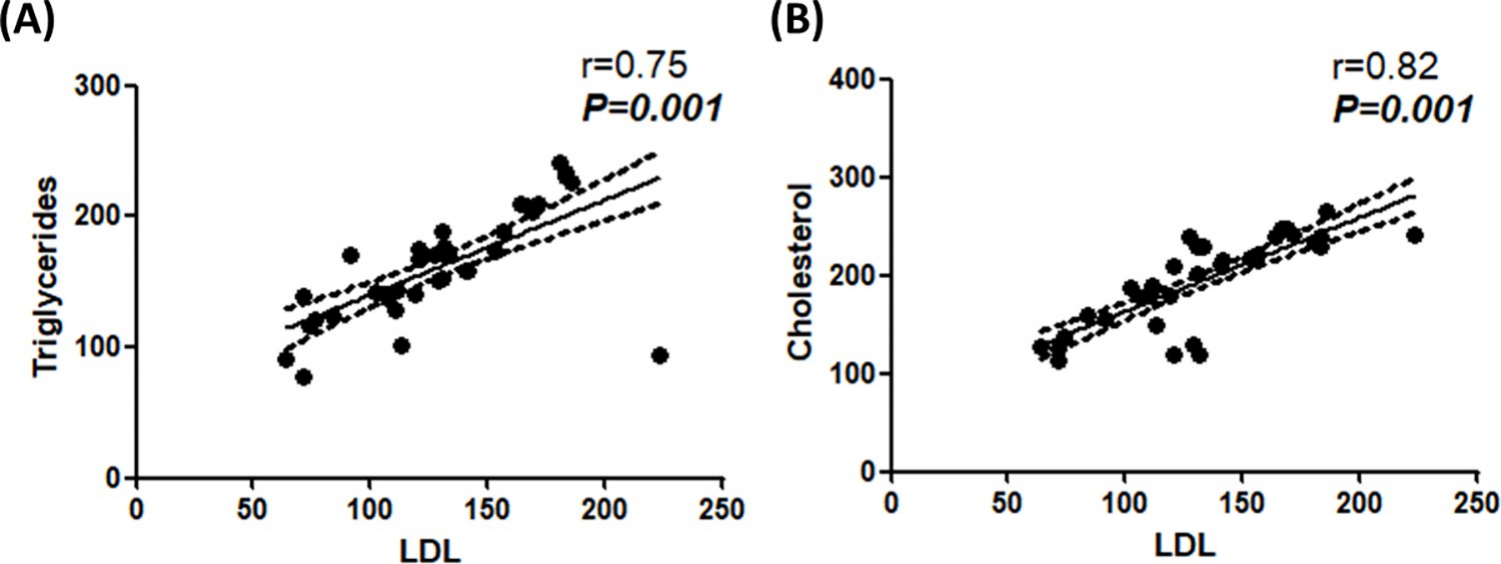
Significant strong correlations between LDL with triglycerides (A) and LDL with cholesterol (B). Correlations between tested markers were performed using spearman correlation.

To test for correlations Spearman correlation analysis was used and as presented in Fig 8, strong correlations were detected between LDL with TG (Fig 8A) and LDL with cholesterol (Fig 8B) at p value of 0.001.

### Overall survival analysis of patients stratified according to their baseline levels of ALP, PSA, CAV1, SLDLRP1 and response to treatment (Fig 9)

**Fig 9:**
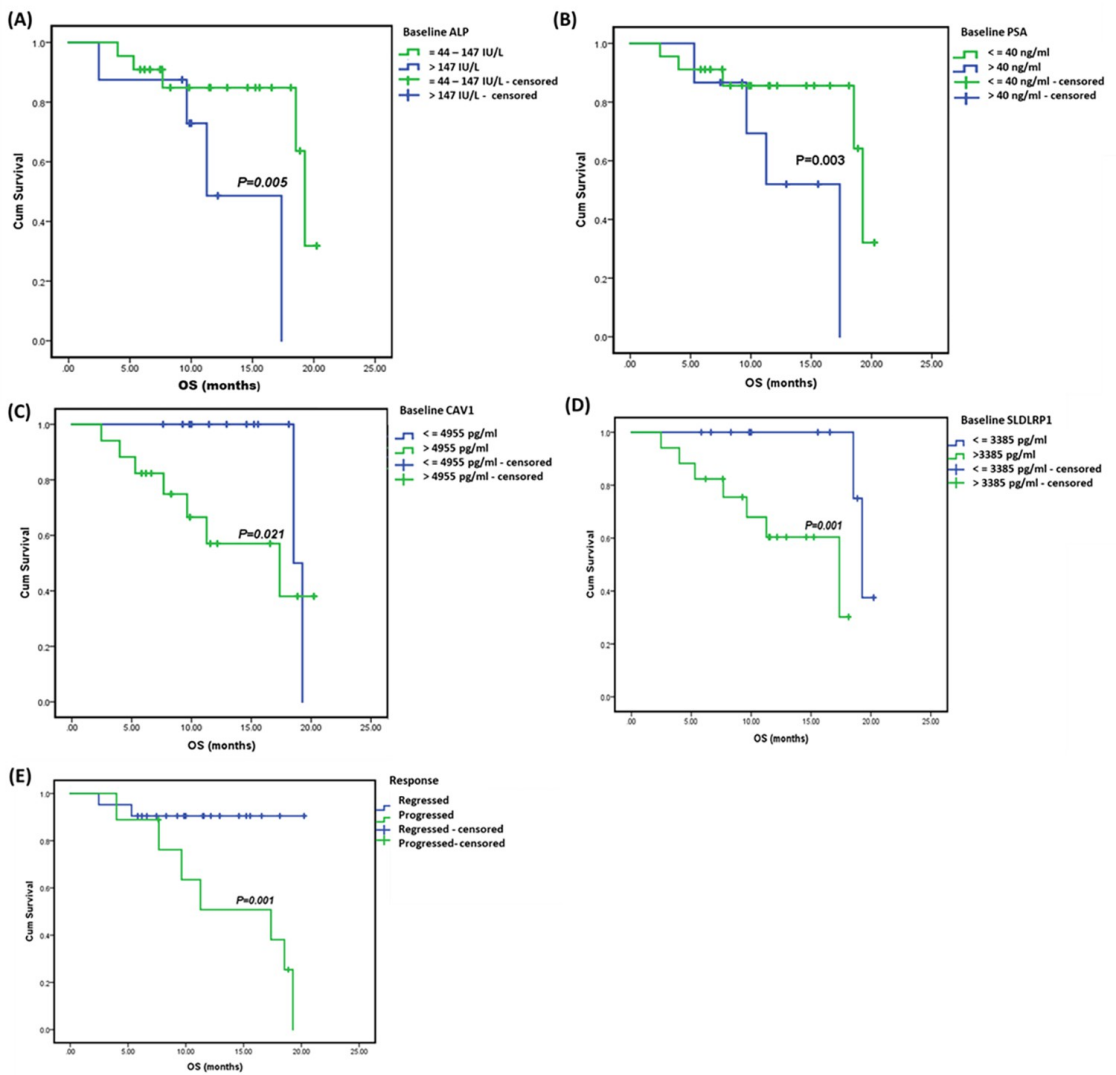
Median rates of overall survival for PC patients stratified according to their baseline levels of ALP (A), PSA (B), Caveolin-1 level (C), SLDLRP1 (D) and response to treatment (E). Median rates of overall survival for PC patients were analysed by Log-Rank test.

The median rates of the overall survival (OS) of PC patients are presented in Fig 9, as shown in Fig 9A the OS was significantly lower in patients with higher baseline ALP level (> 147 IU/L) (p = 0.005). Fig 9B shows that PSA level (<40 ng/ml) was associated with median OS of 17.4, versus 13.27 months (p = 0.003). Fig 9C shows that significantly longer overall survival was recorded in patients with low baseline CAV1 level, <4955 pg/ml, (median OS = 18.9, versus 14.14 months, p = 0.021). Fig 9D shows that lower SLDLRP1 (<3385 pg/ml) was associated with median OS of 19.27, versus 17.37 months (p = 0.001). Fig 9E demonstrates that the OS was significantly lower in patients with progressive course of disease in response to treatment (p = 0.001).

### Hazard ratio for death (Fig 10)

**Fig 10:**
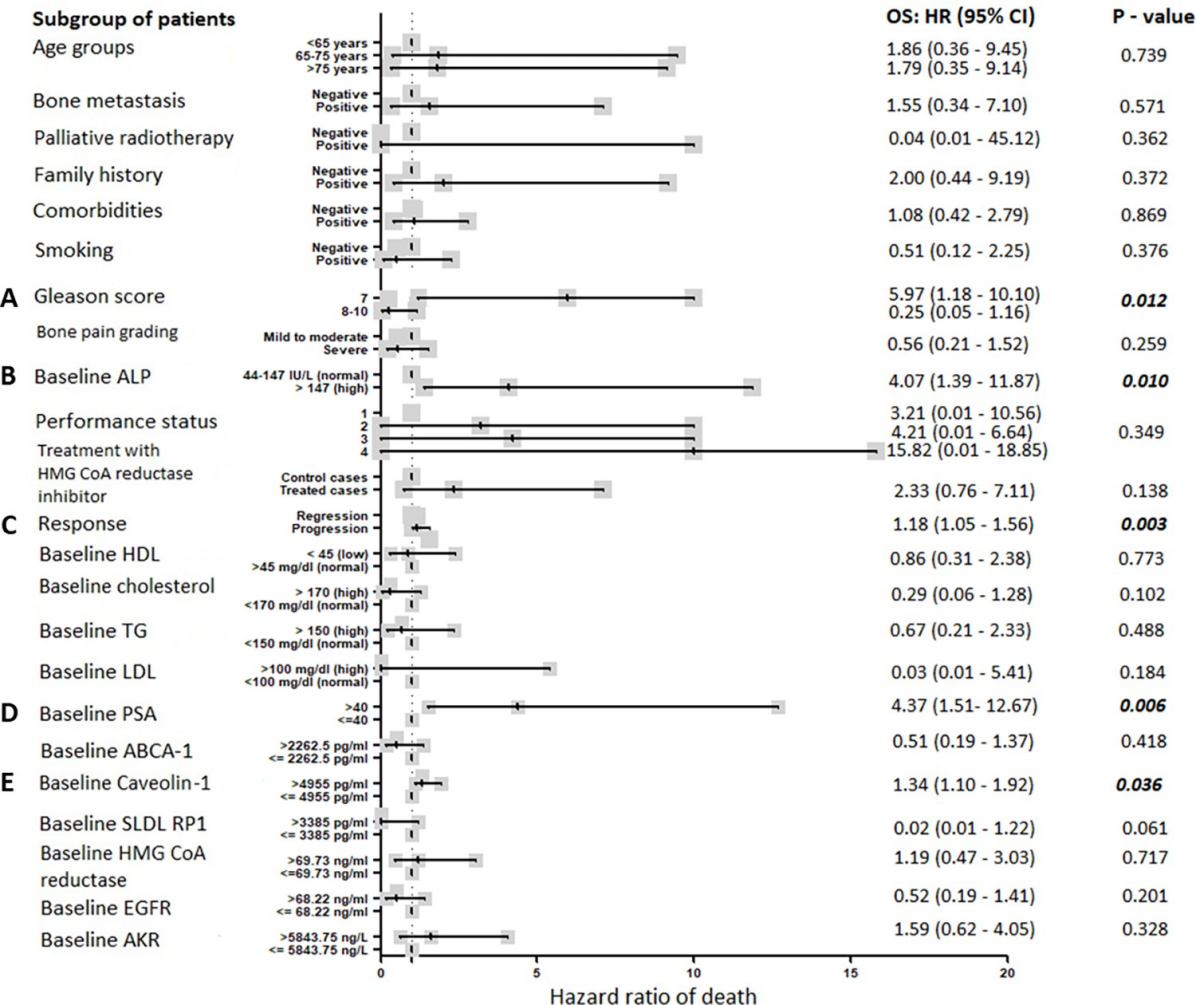
Hazard ratio of death for PC patients stratified according to their Gleason score (A), ALP (B), response to treatment (C), PSA (D) and Caveolin-1 (E). Hazard ratio of death was analysed by COX regression hazard model. Significant p- values are bolded.

Hazard ratio for death Fig 10; was highest with: Gleason score (Fig 10A) (p = 0.012), baseline ALP >147 IU/L (p = 0.010) (Fig 10B), disease progression (p = 0.003) (Fig 10C), baseline PSA >40 ng/dl (p = 0.006) (Fig 10D) and baseline Caveolin-1 >4955 pg/ml (p = 0.036) (Fig 10E).

## Discussion

A recent meta-analysis from observational studies including approximately 1 million patients showed that the use of statins either before or after diagnosis are favorable for both overall survival (HR 0.81, 95% CI 0.72–0.91) and cancer-specific survival (HR 0.77, 95% CI 0.66–0.85) [19]. Previous studies reported that Statins inhibit PC cellular growth and progression *via* cholesterol-mediated and non-cholesterol-mediated pathways. Statins proved to inhibit PC angiogenesis [20] and cellular proliferation [21]. Moreover, inhibition of HMGCR decreases mevalonate and isoprenylated intermediates concentration which support tumor progression [22].

Results of the current study demonstrated that LDL, TG and TC were significantly lower after 6 month of treatment in statin users compared to non-statin users (Fig 2). Moreover, strong positive correlations were detected between the three mentioned lipid profile parameters (Fig 8). Additionally, higher levels of LDL, TG and TC significantly associate with positive PC family history, performance status 3, requirement of palliative radiotherapy, Gleason score > 7 and mortality (Fig 5). These associations indicate the negative impact of increased LDL, TG and TC. Also, LDL level was significantly higher compared to its baseline level in statin non-users; highlighting the fact that castration/androgen deprivation disturb the lipid profile. Our results are consistent with earlier studies which have reported that androgen deprivation was associated with increased LDL, TG and TC [11]. Prostate cancer cells predominantly use fatty acids as energetic substrates to support cellular proliferation [23]. Androgens were proved to stimulate lipolysis and induce the expression of cell surface proteins that control lipids uptake in prostate cancer cells (LNCaP) [24]. This can explain the currently observed increase in LDL following castration; where decreased androgen level was reflected as an increase in LDL level. Thus, in our study the observed lowering effects of Rosuvastatin on LDL, TG and TC can counteract the negative impact of androgen deprivation on lipid metabolism.

Although HMGCR is the target of Rosuvastatin, our results showed that HMGCR was significantly increased in statin users recording 78% higher median level, and decreased in statin non-users after 6 months (Fig 3A). These findings were in contrast to the observed lowering effects of Rosuvastatin on LDL, TG and TC (Fig 2). Interestingly, high levels of baseline HMGCR was associated with age < 65 years, negative bone metastasis and performance status (Fig 6A). Therefore, it remains unclear how Rosuvastatin treatment decreased LDL, TG and TC although it increased HMGCR in our patients. A possible explanation for this is that cholesterol homeostasis is maintained by LDL receptors (LDLR) and HMGCR; [25] which are produced as a result of Sterol Regulatory Element Binding Proteins (SREBPs) activation [26, 27]. Moreover, statins inhibition to HMGCR reduces androgen production and their use at the time of androgen deprivation is associated with delayed progression of PC [13]. Previous studies observed that long-term HMGCR regulation may be affected by aging; due to age-related changes in hormone levels and sensitivity [28–30], justifying the current observed association with age. Heemers and colleagues proved that androgens activate SREBPs to promote de novo lipogenesis and lipid uptake [31]. This can justify the currently observed reduction in HMGCR level due to castration. In agreement with the current results, it was previously reported that HMGCR mRNA levels were upregulated in DU-145 cells with direct proportionality with statin (including rosuvastatin) concentration in the growth medium, but not HMGCR protein expression levels. Inhibition of HMGCR prompts a homeostatic feedback reaction to up-regulate the mevalonate pathway. Blocking HMGCR wash-out the mevalonate pathway endproducts, leading to the activation of SREBPs. Activated SREBPs prompt the transcription of HMGCR and LDLR. Up-regulated LDLR internalizes extracellular LDL and cholesterol [32]. In harmony with the current results, it was previously documented that HMGCR levels are maintained through a feedback response that upregulates both HMGCR mRNA and LDL receptors enabling cholesterol uptake and altered HMGCR protein is not a proof of achieving cholesterol homeostasis even in response to statins that trigger an anti-proliferative effect [33–35].

Our results showed that SLDLRP1 was significantly higher (33%) at 6 month in statin users compared to its baseline level (Fig 3B); which could be explained by statin induction of LDL receptors; this might contribute to increased cholesterol clearance leading to reduced availability of androgens to PC cells. LDL receptor has a crucial impact on statin serum cholesterol reducing effect. In agreement, it was previously reported that Statins upregulate the LDL receptor expression leading to higher LDL clearance from the bloodstream. Normally, LDL receptor expression depends on the level of cholesterol inside the cell [36], while some tumor cells lack feedback regulation of LDL receptor, providing more energy for tumor growth [37]. In addition, previous research found that statin induced inhibition of PC cellular growth was a result of decreased intracellular cholesterol. Higher statin efficacy was in androgen-independent PC where loss of regulation of LDL receptor expression is proved. LDL receptor is reported to have a crucial impact on statin-induced inhibition of PC cellular proliferation [38]. In this context, statins blood cholesterol lowering effect through upregulation of LDL receptor [39] might explain the current observation. Furthermore, higher median level of SLDLRP1 was associated with negative family history of PC (Fig 6D); patients with low baseline SLDLRP1 (< 3385 pg/ml) had a significantly longer overall survival of two months at p-value 0.001 (Fig 9D). Several studies documented the association between SLDLRP1 and various types of cancers [40]. Song and colleagues reported that SLDLPR1 increases the expression of matrix metalloproteinases 2 and 9 in human glioblastoma U87 cells [41]. Additionally, SLDLRP1 was documented to inhibit apoptosis as well as promote tumor proliferation and metastasis, but it possesses dual role in tumor cell migration highlighting that its tumor cell-specific activity needs further research to be clarified [40]. This might explain the current observed better outcome with lower SLDLRP1 level which might be attributed to reducing tumor migration.

Results presented here showed time-dependent increase in AKR1C4 level in both study groups with double percent increase in statin non-users (34% versus 17%, Fig 3C). Moreover, higher median level was associated with negative family history and regressive course of disease appearing to be a favorable marker, but unfortunately association was detected with Gleason score > 7 (Fig 6C). All isoforms of AKR1 are expressed in the liver, but AKR1C4 is the liver specific and it is the most efficient isoform in reducing androstenedione (5α-DHT). This liver specificity suggests its central role in hepatic steroid metabolism. Incubation with AKR1C4 converts DHT to a mixture of 3α-adiol and 3β-adiol [42]. It also catalyzes the reduction of 5α-pregnane-3,20-dione yielding 3α-hydroxy-5α-pregnan-20-one (allopregnanolone) which is the precursor of androsterone [43]. AKR1C4 plays a crucial role in androgen formation by the 5α reduction taking place at the pregnaned level [44, 45]. Moreover, 3β-diol might promote apoptosis in androgen target tissues as the prostate [46]. Circulating testosterone is metabolized in the liver either by 5α-reductase or 5β-reductase yielding 5α-DHT and 5β-DHT, respectively. Subsequent reduction of 3-ketosteroid by AKR1C produces four stereoisomeric tetrahydrosteroids that could be conjugated by phase II enzymes. AKR1C4 possess the highest catalytic efficiency for 5α-DHT-17β-glucuronide or sulfate reduction, thus it is the most efficient in inactivating circulating steroid hormones and limiting their activity [47, 48]. In the liver, AKR1C4 catalyze sequential reactions in chenodeoxycholic and cholic acids biosynthesis from precursors 7α-hydroxy-4-cholestan-3-one and 7α,12α-dihydroxy-4-cholestan-3-one; it catalyzes 3-keto group reduction to 3 α-hydroxy-group [44]. It converts 3-ketosteroids to 3α-hydroxysteroids [49]. This can explain the observed simultaneous increase in AKR1C4 and decrease in TC. This currently observed upregulation of AKR1C4 may explain the noticed reduction in TC level. It was demonstrated *via* a microarray analysis of prostate adenocarcinoma that AKR1C4 gene was altered in 25% of metastatic CRPC cases [48]. The increased genetic expression of AKR1C was correlated with an obvious reduction in the overall survival of CRPC patients. Furthermore, upregulation of AKR1C1, AKR1C2 and AKR1C4 in m PC was reported; where all AKR1C genetic expression was co-regulated and negatively correlated with AR targeted genes expression [50, 51]. In contrast, the current observation associated AKR1C4 with regression of mPC in response to treatment, although rosuvaststin hindered its increase compared to statin non-users no significant effect was reflected on overall survival.

Data presented in our study showed that ATP-binding cassette transporter A1 median level increased with time in both study groups (Fig 3D). Cruz *et al*., reported that prostate cancer cells show increased intracellular cholesterol levels, with loss of ABCA-1-mediated cholesterol efflux [52]. Fukuchi and colleagues documented that ABCA-1 was 20-fold higher in androgen-dependent compared to androgen-independent LNCaP human PC cells, reflecting a probable relationship between ABCA-1 expression levels and PC progression. Also, they proved that androgen suppressive effect on ABCA-1 expression might be one of the mechanisms by which androgens control PC cellular proliferation [53]. This can explain how lowering androgen level by castration increased ABCA-1 level in our study cohort (Fig 3D). We also found that higher ABCA-1 level was associated with negative family history and absence of comorbidities which might be due reducing TC, but surprisingly association was found with performance status 4 (Fig 6B). Reverse cholesterol transport (RCT) is a metabolic pathway that removes tissue excess cholesterol to the liver for elimination; the main player in RCT is ABCA-1 [54]. ABCA-1 is also an essential transporter in the biogenesis of HDL in the liver and the prevention of excess cholesterol accumulation through stimulating the efflux of cholesterol from peripheral tissues [55]. Recent epidemiologic reports showed that low serum TC level and statin use are associated with a lower risk of developing advanced PC, indicating cholesterol impact on aggressive PC development by acting as a substrate for the de novo androgen synthesis. Moreover, marked ABCA-1 downregulation was reported in PC with an inverse correlation with Gleason score. Thus, downregulation of ABCA-1 results in high cholesterol levels inside the cells, leading to tumor progression [56]. Frequent down-regulation of the ABC transporter genes was documented in PC [57]. This can justify the currently observed reduction of TC in both study groups which was more pronounced in the treatment group due to the dual impact of increased ABCA-1 and inhibition of HMGCR. It is important to mention that a previous study documented that androgen suppressive effect on ABCA-1 expression might be one of the mechanisms by which androgens control PC cellular proliferation. Attenuated ABCA-1 expression in androgen-independent cells thus may contribute to PC progression [50]. This can explain the current observed increase (46%) of ABCA-1 median level with time due to androgen deprivation. Oram and Lawn, suggested that ABCA-1 might inhibit the absorption of dietary cholesterol; and drugs that induce ABCA-1 increase cholesterol clearance from tissues and inhibit intestinal absorption of dietary cholesterol [58]. This can illustrate the simultaneous reduction of TC and increase of ABCA-1 in statin users.

In mPC patients, our results showed a significant time-dependent increase in Caveolin-1 in both groups although no significant difference was recorded between statin users and non-users after 6 months, CAV1 level increased by 36% (p = 0.035) at 6 months compared to baseline in the non-statin user group compared to only 9.5% increase in statin users (p = 0.003); thus rosuvastatin hindered CAV1 increase by 26.5% in favor of statin users (Fig 4B), and lower level was associated with longer overall survival from 14.1 to 18.9 months (p = 0.021, Fig 9C). This was confirmed by univariate cox regression analysis where the hazard ratio for death was 1.34 (1.10–1.92, p = 0.036) with higher level (Fig 10E). Additionally, it was reported that smoking increases CAV1 expression [59], in harmony smokers included in this study had higher baseline median CAV1 level (p = 0.017) (Fig 7C). It is well proved that CAV1 is highly associated with the clinical stage and pathological grade of PC, but not with serum PSA levels [60]. Also, it is correlated with the incidence, progression, and metastasis of PC [61]. Caveolin-1 is a key player in prostatic carcinogenesis, metastasis and angiogenesis [62]. Moreover, in mCRPC it might induce radio- and chemo-resistance [63]. It is also crucial for cholesterol transmembrane transport, accumulation and efflux; as it is the primary component of caveolae. In contrast with the current results, previous studies found that statin decreased the expression of CAV1 emphasizing a probable correlation between statins and CAV1 also suggesting a potential pleiotropic value of statins beside lipid reduction [64]. In contrast another study reported that statin increased CAV1 expression, but decreased the density of caveolae while the overall raft density was unaffected. Targeted inhibition of the post mevalonate pathway might be of value in specific reduction of caveolae-based signaling in tumor cells [65]. Previous studies documented that CAV1 binding to cholesterol drives the formation of caveolae. Also, it was found that the level of cholesterol inside the cells is closely related to the expression and activity of CAV1/caveolae. High intracellular cholesterol level stimulates CAV1 expression, and CAV1 drives the cholesterol efflux to caveolae in response to high level of cholesterol inside the cells. Thus, CAV1 might contribute in regulating caveolae and lipid membrane rafts. Additionally, CAV1 expression might be controlled by cholesterol and vice versa [66].

PSA elevation is a result of prostatic architecture disruption in prostate diseases and serum levels correlate with cancer stage. It is worth mentioning that poorly differentiated PC produces less PSA than well-differentiated ones [67]. The tumor marker PSA showed a significant drop with time due to castration in both groups (Fig 4A). Higher level was associated with shorter overall survival of 13.27 compared to 17.4 months at P- value 0.003 (Fig 9B); confirmed by the hazard ratio for death with higher level of PSA was 4.37 (1.51–12.67) at P- value 0.006 (Fig 10D). Moreover, higher level of PSA was significantly associated with positive bone metastasis and consequently high level of ALP at p values 0.013 and 0.002, respectively (Fig 7A). Previous studies reported that high Gleason scores and elevated PSA levels are associated with bone metastasis in PC patients [68, 69]. Also, it was found that statins lower PSA levels, but with limited clinical significance [70]. In contrast another study reported a statistically significant decline in PSA levels following statin initiation; with the most pronounced reduction among patients with the largest LDL declines [71]. Unfortunately, in the current study no significant difference was detected between the two study groups in PSA level at any time point and no tangible correlation was proved between PSA and LDL.

Previous research found that elevated serum ALP is associated with poor overall survival and progression free survival in PC and reported that ALP is a reliable marker for PC prognosis. Also, it is a valuable indicator for bone metastatic load [72]. In harmony, results of the current study showed that the bone marker ALP higher baseline level was associated with positive family history and receiving palliative radiotherapy to bone metastasis (Fig 7B). Another study showed that ALP baseline level is associated with overall survival and a superior prognostic marker than PSA in bone-dominant mCRPC [73, 74]. In agreement, this study found that the hazard ratio for death was 4.07 (1.39–11.87) at P- value 0.010 (Fig 10B); patients with higher baseline level (> 147 IU/L) showed shorter overall survival of 11.27 compared to 19.27 months at P- value 0.005 (Fig 10A). One of the drawbacks in this study was the inability to measure the level of ALP over time, it was only measured at baseline; thus, no observation was reported about the effect of rosuvastatin on ALP level in our cohort.

In the current study, EGFR showed initial increase at 3 months followed by reduction at 6 months to record a significantly lower level in statin users compared to non-statin users (Fig 4C). Higher level was correlated with positive family history, presence of comorbidities, mild to moderate bone pain and performance status 4 (Fig 7D). Earlier studies elaborated that EGFR localize within lipid rafts and its signaling pathway depend on the lipid-rafts cholesterol content [75]. Additionally, disrupting the lipid rafts through cholesterol depletion interferes with the receptor activation, leading to inhibition of cell growth and development [76]. Moreover, EGFR is crucial in the resistance to hormonal therapy [77]. This might explain the current results; where rosuvastatin decreased TC level, disrupting the lipid rafts leading to the more pronounced EGFR reduction in statin users. Previous studies reported that androgens increase EGFR mRNA levels and inhibition of EGFR prevents androgen driven proliferation [78]. Treatment of PC cells with cholesterol lowering agents can disrupt lipid raft organization and interfere with EGFR signaling [79]. Epidermal growth factor receptor is vital for PC development and proliferation [80]. Several suggested mechanisms, including EGFR, intratumoral androgens, AR alterations, might contribute to maintaining AR signaling during androgen deprivation. Due to the observed importance of EGFR in PC progression its inhibition might overcome CRPC [81–83]. It was previously documented that statins inhibited EGFR autophosphorylation and its downstream signaling pathway [84]. Thus, the observed statin induced reduction in EGFR might contribute to the beneficial effect of statin use in PC patients.

In conclusion, the current study suggests that in Egyptian mPC patients, Gleason score, baseline ALP might be combined with periodic assessment of PSA, CAV1 and SLDLRP1 levels; to predict patients’ prognosis and overall survival. Additionally, statin (Rosuvastatin) might modify mPC outcome through modulatory lowering effect of the lipid profile and downregulation of EGFR. Undeniably, limitations of the current study include the limited number of patients and short follow up duration, but being a randomized prospective controlled study is a favorable point as we could assess patients’ compliance, as well as limit selection bias. Also, clinical and molecular outcomes were both assessed. Moreover, the NCI of Egypt receives patients from all over Egypt; so the population, although small is not limited to one area. The clinical significance is that Cholesterol, triglycerides, LDL as well as EGFR levels may be related to PCa severity and might be of relevance to patient management and prognosis, highlighting that assessment of these parameters should be routinely evaluated. To conclude, there is a need for extending this trial on a larger cohort from multicenters with a longer follow-up period to properly validate the effect of intervention.

## Supporting information

S1 Checklist CONSORT 2010 checklist of information to include when reporting a randomised trial*. (PDF)

S1 Data (XLSX)

S1 File (PZF)

S2 File (PZF)

S1 Protocol (DOCX)
