# Supplementary material for: The prognosis of lipid reprogramming with the HMG-CoA reductase inhibitor, rosuvastatin, in castrated Egyptian prostate cancer patients: Randomized trial
Source: PLoS One. 2022 Dec 8;17(12):e0278282. doi: 10.1371/journal.pone.0278282 (PMC9731457; doi:10.1371/journal.pone.0278282)
Supplement: S1 Protocol — (DOCX) [file pone.0278282.s005.docx]

| **National cancer Institute, Cairo University postgraduate Research Protocol From** | | |
| --- | --- | --- |
| **1-Proposed study Title**: Therapeutic Role of HMG-CoA Reductase Inhibition in Castrated Egyptian Prostate Cancer Patients | | |
| **2- Candidate Name:** Riham Mohamed Abdel Maksoud Karkeet | | |
| **3- Date of Registration:** 8 December 2018 | | |
| a- Occupation: Assistant lecturer of Pharmacology and Experimental Oncology - Cancer Biology Department - National Cancer Institute -Cairo University | | |
| riham.karkeet@nci.cu.edu.eg | | b- E-mail address: |
| 01220112453 | | c- Phone number: |
| **4-Investigators Contact Information:** | | |
| Prof. Sherif Yehia Saad | a-Principle investigator: | |
| Prof. Abdelrahman N. Zekri  Prof. Ghada M. Sherif  Prof. Mohamed M. Sayed-Ahmed  Prof. Salem E. Salem  Dr. Ahmed Abdelbary  Dr. Mariam Fouad | b. other investigators: | |
| Cancer Biology | c- Department's name: | |
| riham.karkeet@nci.cu.edu.eg | d- E-mail address: | |
| 01220112453 | e- Phone number: | |

| **5- Background and Rationale:**  Globally, prostate cancer (PC) represents the second most frequently diagnosed malignant disease and the sixth leading cause of cancer related death among men and its burden is almost expected to be doubled by 2040 due to population growth and aging [^[[1]](#endnote-1)^]. In Egypt, PC represents 4.5% of male malignancies, but with apparently low incidence and high mortality due to higher prevalence of advanced and metastatic disease [^[[2]](#endnote-2)^,^[[3]](#endnote-3)^]. The treatment of advanced PC is basically dependent on the initial effective responses with androgen deprivation therapy (ADT), through which patients’ progress over time to metastatic castration-resistant prostate cancer (mCRPC). Strategies developed to counteract ADT resistance have had only modest clinical benefit [^[[4]](#endnote-4)^,^[[5]](#endnote-5)^]. Nevertheless, relapsed or metastatic disease following castration has a poor prognosis with most patients dying within two years [^[[6]](#endnote-6)^,^[[7]](#endnote-7)^,^[[8]](#endnote-8)^,^[[9]](#endnote-9)^,^[[10]](#endnote-10)^].  The metabolic effects of ADT, including disturbance of lipid profile leading to increased risk for diabetes, metabolic syndrome and cardiovascular morbidity/mortality may have a role in treatment related morbidity in these patients [^[[11]](#endnote-11)^].  Lipid metabolism is strikingly affected by androgens and dysregulation of lipid metabolism is a key feature of PC development. Androgens may stimulate *de novo* lipogenesis and lipid uptake. HMG coA reductase inhibitors (Statins) are administered widely worldwide for their clinical expressive lipid-lowering effect. Statins not only prevent the production of cholesterol but also block the formation of many intermediary lipid molecules, which might function as cellular signaling pathways stimulant, provoking the major function of cholesterol synthesis in accelerating progression to CRPC.  Several epidemiological reports have shown significant associations between statin use and decreased incidence of advanced PC, risk of recurrence after local treatment, mortality, and PSA levels relative to Statin nonusers [^[[12]](#endnote-12)^,^[[13]](#endnote-13)^].  Statins decrease advanced stage prostate cancer risk raising a possible correlation between cholesterol and prostate cancer risk [^[[14]](#endnote-14)^].  Moreover, high Cholesterol levels have proved to increase the risk of aggressive PC, and according to Jiang and colleagues, statin users after prostatectomy had a less aggressive disease [^[[15]](#endnote-15)^]. Rosuvastatin is the most powerful widely‐prescribed statin worldwide; due to its rapid absorption, achieving peak plasma concentration within three hours. Moreover, its lipid‐lowering effect is not influenced by the time‐of‐day by which it is administered; due to the comparatively long half‐life of 20 hours [^[[16]](#endnote-16)^,^[[17]](#endnote-17)^]. |
| --- |
| **6- Objectives:**  This study aims to investigate, on mechanism-based, the possible therapeutic effects of rosuvastatin when combined with androgen deprivation in metastatic prostate cancer patients. |
| **7- Study Design :**  Prospective study, a patient cohort will be selected according to the following criteria (According to the institutional review board of the NCI): Newly diagnosed cancer prostate patients over 50 years old who will be under primary intervention with surgical castration. Statin will be used at the time of castration. Patients will be classified into two groups treated with castration with or without Rosuvastatin 20 mg/day. |
| **8- Study Methodology:**   1. Population of study &disease Condition   A cohort of 80 patients will be selected according to the following criteria (According to the institutional review board of the NCI): Newly diagnosed cancer prostate patients over 50 years old who will be under primary intervention surgical castration. Statin will be used at the time of castration. Patients will be classified into two groups treated with castration with or without Statin. Using power 85% and 5% significance level, 35 patients are required. |
| b) Back ground and Demographic characteristics   - Male - Over 50 year old   Metastatic prostate cancer |
| c) Inclusion criteria:  Naïve newly diagnosed with metastatic prostate cancer  ▪ Age ≥ 50 years.  ▪ No psychological or geographical barriers for regular follow up of the patients. |
| d) Exclusion criteria:  ▪ Age < 50 years.  ▪ psychological or geographical barriers for regular follow up of the patients. |

| e) Interventions:  Rosuvastatin 20 mg/day for 6 months will be administered at the time of castration | |
| --- | --- |
| f) Possible Risk: **No** drug interaction |  |
| g) Primary outcome parameter   - Lipid profile and metabolism markers: LDL, cholesterol, triglycerides, HDL, HMG-CoA reductase, ABCA1 , SLDLRP1, AKR1C4. - Tumor aggressiveness markers: PSA, ALP, Caveolin-1, EGFR |  |
| h) Secondary outcome parameters:  Short term clinical outcome in terms of disease progression/regression and overall survival |  |
| i) Sample size  A cohort of 80 patients will be selected according to the following criteria (According to the institutional review board of the NCI). Patients will be classified into two groups treated with surgical castration with or without Statin. |  |
| j) Statistical analysis:  Randomization, data management and statistical analysis were performed using The Statistical Package for Social Sciences (SPSS) version 24. Normal distribution and variance homogeneity of data were assessed using the Kolmogorov–Smirnov and Levene’s tests, respectively.  Numerical data were summarized using median and interquartile range (IQR). Categorical data were summarized as count and percentage. Patients were stratified according to their clinicopathological factors and for more than two subgroups of patients, the change in measured parameters were tested for significance using Kruskal–Wallis test and the pairwise comparison were done using Mann–Whitney. The change in proteins concentration over time was tested using Friedman test of significance. Spearman correlation analysis was used to test all possible correlations. Kaplan- Meier survival analysis was used to calculate the cumulative survival rate as well as median levels of OS after two years of follow up. OS was calculated from date of diagnosis to date of death by any cause. Living patients or patients lost to follow-up were censored on the last known alive date. The hazardous effect of death or progression Cox proportion hazard Model was used to evaluate the hazardous effect of different clinicopathological and proteins levels on death and progression. All P-values are two-sided. P-values < 0.05 were considered significant. Patients were equally allocated between the two study arms, but ten patients from the control group lost follow-up and were excluded from all tests. |  |
| k) Source of funding: Cairo University |  |

| **9-Ethical committee approval:**  The study ID BB1901-30303 was approved by the Institutional Human Research Ethics Committee of NCI, Egypt, Number 00004025, with IRB review Number 201819019.3 |
| --- |
| **10- Cooperation with other departments:**  Name of department : Medical Oncology  Name of investigator: Salem E. Salem  Name of department : Surgical Oncology  Name of investigator: Ahmed Abdelbary  Name of department : Biostatistics and Cancer Epidemiology  Name of investigator: Ghada Sherif |

1. [] Ferlay J, Ervik M, Lam F, Colombet M, Mery L, Piñeros M, et al. Global cancer observatory: cancer today. International Agency for Research on Cancer, Lyon, France, 2018. <https://gco.iarc.fr/today>  [↑](#endnote-ref-1)
2. [] Elabbady A, Eid A, Fahmy A, Kotb AF. Pattern of prostate cancer presentation among the Egyptian population: A study in a single tertiary care center. Central European Journal of Urology. 2014; 67(4):351-356. DOI: [10.5173/ceju.2014.04.art](https://doi.org/10.5173/ceju.2014.04.art7) [↑](#endnote-ref-2)
3. [] Rabah DM, Arafa MA. Prostate cancer screening in a Saudi population: an explanatory trial study. Prostate Cancer Prostatic Dis. 2010; 13(2):191-4. DOI: [10.1038/pcan.2009.60](https://doi.org/10.1038/pcan.2009.60) [↑](#endnote-ref-3)
4. [] Small EJ, Halabi S, Dawson NA, Stadler WM, Rini BI, Picus J, et al. Antiandrogen withdrawal alone or in combination with ketoconazole in androgen-independent prostate cancer patients: a phase III trial (CALGB 9583). J. Clin. Oncol. 2004; 22, 1025–1033. DOI: [10.1200/JCO.2004.06.037](https://doi.org/10.1200/jco.2004.06.037) [↑](#endnote-ref-4)
5. [] Yap TA, Zivi A, Omlin A, de Bono JS. The changing therapeutic landscape of castration-resistant prostate cancer. Nat Rev Clin Oncol. 2011; 8(10):597-610. DOI: 10.1038/nrclinonc.2011.117. PMID: 21826082. [↑](#endnote-ref-5)
6. [] Chandrasekar T, Yang JC, Gao AC, Evans CP. Mechanisms of resistance in castration-resistant prostate cancer (CRPC). Transl Androl Urol. 2015; 4(3):365-380. DOI: [10.3978/j.issn.2223-4683.2015.05.02](https://doi.org/10.3978/j.issn.2223-4683.2015.05.02) [↑](#endnote-ref-6)
7. [] Sampson N, Neuwirt H, Puhr M, Klocker H, Eder IE. In vitro model systems to study androgen receptor signaling in prostate cancer. Endocrine-Related Cancer. 2013; 20: R49- R64. DOI: [10.1530/ERC-12-0401](https://doi.org/10.1530/erc-12-0401) [↑](#endnote-ref-7)
8. [] Penning TM. Androgen biosynthesis in castration-resistant prostate cancer. Endocrine-Related Cancer. 2014; 21: T67- T78. DOI: [10.1530/ERC-14-0109](https://dx.doi.org/10.1530%2FERC-14-0109) [↑](#endnote-ref-8)
9. [] Karantanos T, Evans CP, Tombal B, Thompson TC, Montironi R, Isaacs WB. Understanding the mechanisms of androgen deprivation resistance in prostate cancer at the molecular level. Eur Urol. 2015; 67:470–479. DOI: [10.1016/j.eururo.2014.09.049](https://www.researchgate.net/deref/http%3A%2F%2Fdx.doi.org%2F10.1016%2Fj.eururo.2014.09.049?_sg%5B0%5D=k7xZkUlZp5heTvcYDcChH9c01avvTqwjTs3EMHKHBJVfoVxK6uJ7zZ5rzEPaeJdjcxtez9cI0VukHluUpoE2u9I6ZQ.9Ia1ByFzsQ3V0ZEEAvJ0JQ2suLoEkXxiZIHF6AjLPBKwxHeWOnjGu7xuQ6ib9rVEnPudSsR9WfptHRmh5mMAEQ) [↑](#endnote-ref-9)
10. [] Patki M, Huang Y, Ratnam M. Restoration of the cellular secretory milieu overrides androgen dependence of in vivo generated castration resistant prostate cancer cells overexpressing the androgen receptor. Biochem Biophys Res Commun. 2016; 476:69–74. DOI: [10.1016/j.bbrc.2016.05.058](https://doi.org/10.1016/j.bbrc.2016.05.058) [↑](#endnote-ref-10)
11. [] Choi SM, Kam SC. Metabolic effects of androgen deprivation therapy. Korean journal of urology. 2015; 56(1), 12–18. DOI: [10.4111/kju.2015.56.1.12](https://doi.org/10.4111/kju.2015.56.1.12) [↑](#endnote-ref-11)
12. [] Liu DD, Han CC, Wan HF, He F, Xu HY, Wei SH, et al. Effects of inhibiting PI3K-Akt-mTOR pathway on lipid metabolism homeostasis in goose primary hepatocytes. Animal. 2016; 10:1319–1327. DOI: [10.1017/S1751731116000380](https://www.researchgate.net/deref/http%3A%2F%2Fdx.doi.org%2F10.1017%2FS1751731116000380?_sg%5B0%5D=hAhgeEEFBbckeJurGSsJzq6fEXvdOPWnbUtG5hZHh9G0Pj-ARXlNP6LAG3B-gsH_D4ym1FiWJhpPToEN99e36hL9SQ.oboH4_0YI0vXLHDv95uLRzKqRuRFiyYLf173W0DtocLfS75L8JtFycMMYY0MFQTHvP_gzQJypXtTONjetwcyMw) [↑](#endnote-ref-12)
13. [] Harshman LC, Wang X, Nakabayashi M, Xie W, Valenca L, Werner L, et al. Statin Use at the Time of Initiation of Androgen Deprivation Therapy and Time to Progression in Patients With Hormone-Sensitive Prostate Cancer. JAMA Oncol. 2015; 1(4): 495-504. DOI: [10.1001/jamaoncol.2015.0829](https://dx.doi.org/10.1001%2Fjamaoncol.2015.0829) [↑](#endnote-ref-13)
14. [] [Jespersen CG, Norgaard M, Friis S, Skriver C, Borre M. Statin use and risk of prostate cancer: a Danish population-based case-control study, 1997-2010. Cancer Epidemiol. 2014; 38(1):42-47.](https://www.urotoday.com/recent-abstracts/urologic-oncology/prostate-cancer/67122-statin-use-and-risk-of-prostate-cancer-a-danish-population-based-case-control-study-1997-2010-abstract.html) DOI: [10.1016/j.canep.2013.10.010](https://doi.org/10.1016/j.canep.2013.10.010) [↑](#endnote-ref-14)
15. [] Jiang S, Wang X, Song D, Liu X,  Gu Y, Xu Z, et al. Cholesterol Induces Epithelial-to-Mesenchymal Transition of Prostate Cancer Cells by Suppressing Degradation of EGFR through APMAP. Cancer Res. 2019; (79) (12) 3063-3075. DOI: 10.1158/0008-5472.CAN-18-3295 [↑](#endnote-ref-15)
16. [] Goodman LS, Brunton LL, Chabner B, Knollmann BC. Goodman & Gilman's Pharmacological Basis of Therapeutics. 12th Edition. New York: McGraw‐Hill, 2011. [ISBN: 9780071624428] [↑](#endnote-ref-16)
17. [] Adams SP, Sekhon  SS, Wright  JM. Rosuvastatin for lowering lipids. Cochrane Database of Systematic Reviews. 2014; 11(CD010254). DOI: 10.1002/14651858.CD010254.pub2. [↑](#endnote-ref-17)
